# Metabolic profile and differentiation potential of extraembryonic endoderm-like cells

**DOI:** 10.1038/s41420-018-0102-1

**Published:** 2018-09-26

**Authors:** Mohamed I. Gatie, Gregory M. Kelly

**Affiliations:** 1 Department of Biology, Collaborative Graduate Specialization in Developmental Biology, The University of Western Ontario, London, ON Canada; 2 Department of Paediatrics, The University of Western Ontario, London, ON Canada; 3 Department of Physiology and Pharmacology, The University of Western Ontario, London, ON Canada; 4 Child Health Research Institute, London, ON Canada; 5 Ontario Institute for Regenerative Medicine, Toronto, ON Canada

## Abstract

Glucose metabolism has a crucial role for providing substrates required to generate ATP and regulate the epigenetic landscape. We reported that F9 embryonal carcinoma stem-like cells require cytosolic reactive oxygen species to differentiate into extraembryonic endoderm; however, mitochondrial sources were not examined. To extend these studies, we examined the metabolic profile of early and late-passage F9 cells, and show that their ability to differentiate is similar, even though each population has dramatically different metabolic profiles. Differentiated early-passage cells relied on glycolysis, while differentiated late-passage cells transitioned towards oxidative phosphorylation (OXPHOS). Unexpectedly, electron transport chain protein stoichiometry was disrupted in differentiated late-passage cells, whereas genes encoding mitofusion 1 and 2, which promote mitochondrial fusion and favor OXPHOS, were upregulated in differentiated early-passage cells. Despite this, early-passage cells cultured under conditions to promote glycolysis showed enhanced differentiation, whereas promoting OXPHOS in late-passage cells showed a similar trend. Further analysis revealed that the distinct metabolic profiles seen between the two populations is largely associated with changes in genomic integrity, linking metabolism to passage number. Together, these results indicate that passaging has no effect on the potential for F9 cells to differentiate into extraembryonic endoderm; however, it does impact their metabolic profile. Thus, it is imperative to determine the molecular and metabolic status of a stem cell population before considering its utility as a therapeutic tool for regenerative medicine.

## Introduction

Metabolism provides substrates for energy expenditure^1–3^ and can modulate the epigenome, thereby influencing cell fate^4–6^. Typically, somatic cells rely on oxidative phosphorylation (OXPHOS) to generate ATP, whereas proliferative cancer and stem cells use glycolysis^7–11^. ATP requirements in proliferative cells are high and, although OXPHOS is more efficient in generating ATP, sufficient glucose flux in glycolysis compensates for the rate of ATP production^12–14^. This categorization of metabolic profiles is distinct in early mammalian embryos^15^. Naive embryonic stem cells (ESCs) use glycolysis and OXPHOS, whereas primed ESCs, having structurally mature mitochondria capable of OXPHOS, transition from bivalent metabolism to glycolysis^16,17^. Studies show that extraembryonic trophoblast stem cells preferentially use OXPHOS to produce ATP^18^. However, the metabolic profile of extraembryonic endoderm (XEN) stem cells, which differentiate into primitive (PrE) or parietal endoderm (PE) in a process recapitulated using F9 embryonal carcinoma stem-like cells (F9 cells), remains unknown^19–21^. We reported that F9 cells require increased levels of cytosolic reactive oxygen species (ROS) to differentiate into PrE^22–24^, but the role of the mitochondria, a major source of ROS, has not been investigated.

Mitochondria and metabolism have a key role in the reprogramming of somatic cells to induced pluripotent stem cells (iPSCs). These events require a metabolic transition from OXPHOS to glycolysis in order for cells to sustain proliferation and to reset the epigenetic landscape^25–27^. The acquisition of pluripotency is not immediate as iPSCs that have undergone few passages share a molecular and epigenetic signature reminiscent of their somatic counterparts, whereas prolonged passaging resets their profile closer to ESCs^28–30^. However, and although not universal^31,32^, ESCs passaged extensively develop abnormal karyotypes, yet maintain pluripotency and differentiation potential^33^. Although studies have focused on the metabolic status of stem cells or the effects of passaging on their ability to differentiate, an understanding of how the two are linked is limited.

To address this, two populations of F9 cells were investigated and results show that early and late-passage cells had similar differentiation potential, but each have dramatically different metabolic profiles. These differences observed were due to changes in the expression and protein levels of pyruvate dehydrogenase (PDH) kinases (PDKs), which regulate the activity of PDH complex, thereby influencing the metabolic profile of cells. In addition, genes encoding mitochondrial fusion proteins were upregulated in early-passage F9 cells, while relative levels of mitochondrial electron transport chain (ETC) proteins were disrupted in late-passage cells. Surprisingly, culturing either cell population under their preferred metabolic conditions enhanced the exit from pluripotency and promoted PrE formation. More importantly, late-passage cells possessed an abnormal karyotype, resulting in increased proliferation rates, which were correlated to significant increases in the expression of cell cycle regulators. Together, these results demonstrate that early- vs. late-passage F9 cells retain their ability to differentiate into XEN; however, this ability to occur in cells that have different metabolic profiles and chromosomal composition, underpins the importance of monitoring the physiology of stem cell populations to ensure their quality as a tool for regenerative medicine.

## Results

### Late-passage F9 cells differentiate to XEN-like cells

Undifferentiated late-passage F9 cells grew in compact colonies, while those induced to form PrE or PE adopted a stellate-like phenotype (Fig. S1A). *Oct4* expression in RA-induced PrE was similar to controls (Fig. S1B), but protein levels were reduced significantly (Fig. S1F, G). This was more dramatic in cells induced to PE by RA and db-cAMP (RDB; Fig. S1B, F, G). Increased expression of *Gata6* and *Dab2* (Fig. S1C, D, respectively), and levels of DAB2 (Fig. S1F, H) and KERATIN-8 (Fig. S1F, I) were evidence that F9 cells had differentiated into PrE. Finally, significant increases in Thrombomodulin (*Thbd*) expression (Fig. S1E) and in THBD levels (Fig. S1F, J) indicated that cells had differentiated to PE. Collectively, these results support the findings that F9 cells differentiated to PrE and PE-like lineages when treated with RA and RDB, respectively^20,21^.

### Mitochondrial activity during late-passage differentiation

**Fig. 1:**
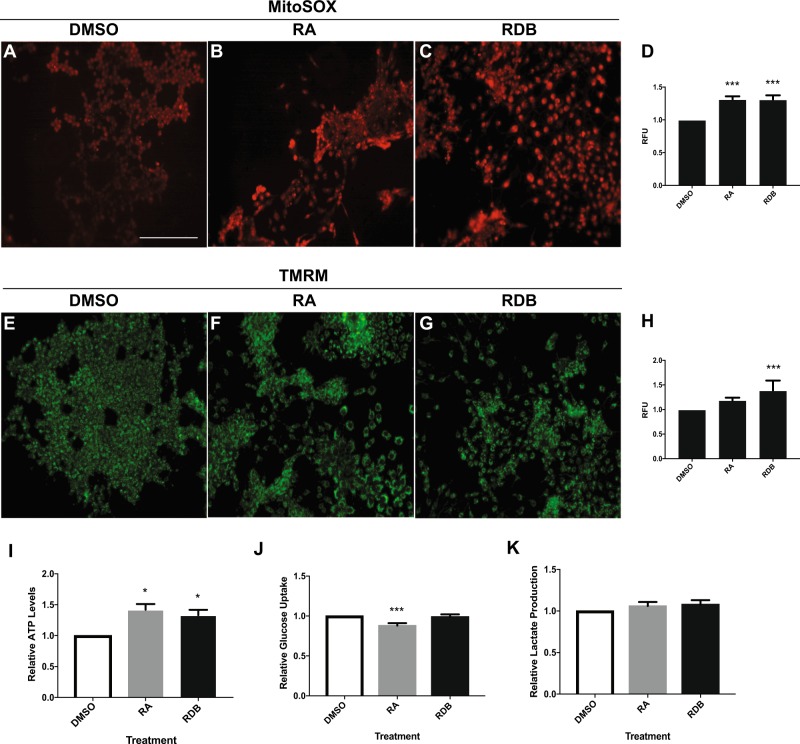
Differentiated late-passage F9 cells display increased mitochondrial ROS production, membrane potential, and ATP levels. **A**–**C** Fluorescence images and quantification of F9 cells (**D**) stained with MitoSOX to detect mitochondrial superoxide in undifferentiated F9 cells, primitive endoderm (RA treatment) and parietal endoderm (RDB treatment). **E**–**G** Fluorescence images and quantification (**H**) of F9 cells stained with TMRM to detect mitochondrial membrane potential during F9 cell differentiation. **I** ATP levels, **J** relative glucose uptake, and **K** lactate production were also measured during differentiation. Values are presented as mean ± SEM of at least three biological replicates. Significance was tested using a one-way ANOVA followed by a Tukey’s test. ^*^P < 0.05, ^***^P *<* 0.001. (Scale bar, 20 μm)

Although ROS generated from cytoplasmic sources accompanies F9 cell differentiation^22–24^, mitochondrial sources were never examined. To address this, late-passage F9 cells were treated with DMSO-, RA-, or RDB, and then stained with MitoSOX to detect mitochondrial superoxide (Fig. 1A–C) or with TMRM for mitochondrial activity (Fig. 1E–G). PrE cells showed a significant increase in mitochondrial superoxide levels relative to controls (Fig. 1D), but not mitochondrial activity (Fig. 1H). Significant differences in superoxide levels and mitochondrial activity were also noted in PE cells (Fig. 1D, H, respectively). These changes suggested that differentiation was accompanied by an increase in OXPHOS, which was supported by the increase in ATP levels in these cells (Fig. 1I). Cells were also assayed for changes in metabolites in the media, and results showed that overall, a significant decrease in glucose uptake (Fig. 1J), but not lactate production (Fig. 1K) occurred in PrE cells. To examine if these changes were due to altered expression of glucose transporters^35^, cells were differentiated, and then assayed for *Glut1*–*4*, *8*, and *9* (Fig. S2). *Glut1*, *3*, and *9* expression increased significantly in PrE, whereas *Glut3* and *9* were significantly upregulated in PE. Conversely, *Glut4* transcript abundance decreased in PrE, whereas *Glut2* and *8* expression remained unchanged. These results suggested that GLUT4 is likely the key glucose transporter during RA-induced differentiation of late-passage F9 cells.

### Metabolic changes accompanying late-passage differentiation

**Fig. 2:**
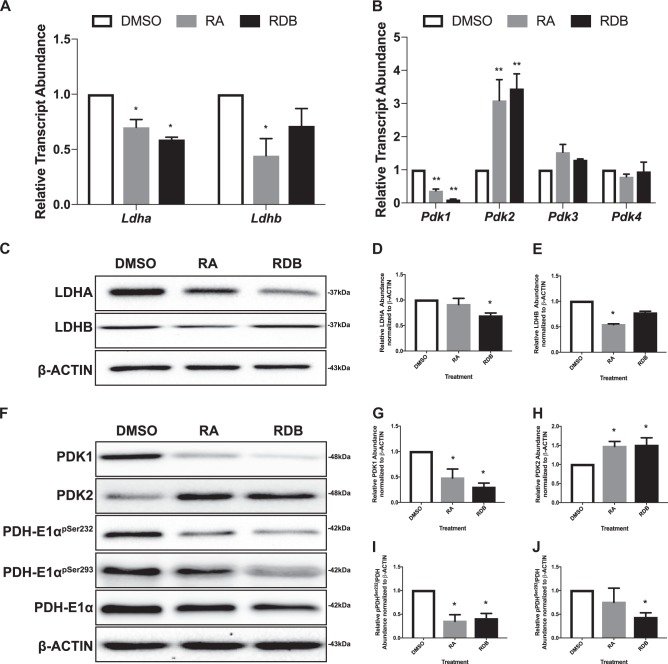
Differentiated late-passage F9 cells downregulate transcripts and enzymes involved in inhibiting OXPHOS metabolism. Relative transcript abundance of **A** *Ldha* and *Ldhb*, and **B** *Pdks* during F9 cell differentiation. *L14* was used a constitutive gene for qRT-PCR. **C** Representative immunoblot and densitometric analyses (**D**, **E**) of LDHA and LDHB, respectively, during F9 cell differentiation. **F** Representative immunoblot showing PDK1, PDK2, PDH-E1α^pSer232^, PDH-E1α^pSer293^, and PDH-E1α levels, and densitometric analysis of **G** PDK1, **H** PDK2, **I** PDH-E1α^pSer232^, and **J**. PDH-E1α^pSer293^ levels. β-Actin served as a loading control. Values are presented as mean ± SEM of at least three biological replicates. Significance was tested using a one-way ANOVA followed by a Tukey’s test. ^*^P < 0.05, ^**^P *<* 0.01

To examine the metabolic profile, late-passage F9 cells were differentiated and then analyzed to detect transcripts encoding Lactate dehydrogenase A (LDHA), which promotes the conversion of pyruvate to lactate, and LDHB, which catalyzes the reverse reaction. *Ldha* expression was significantly downregulated in PrE and PE cells (Fig. 2A), but only in PE cells at the protein level (Fig. 2C, D). However, *Ldhb* transcripts were only significantly downregulated in PrE (Fig. 2A) and a similar trend was seen at the protein level (Fig. 2C, E). The expressions of *Pdk1–4* (Fig. 2B), which encode PDK isoforms that phosphorylate and inactivate the PDH complex, were examined (Fig. 2B). Results show that *Pdk1* was significantly downregulated during differentiation, whereas *Pdk2* was significantly upregulated relative to controls (Fig. 2B). Similar results were seen at the protein level (Fig. 2F, G, H). Despite these increases, PDH-E1α^pSer232^ (Fig. 2F, I) and PDH-E1α^pSer293^ (Fig. 2F, J) levels were reduced significantly, suggesting that PDK1 likely regulates the activity of the PDH complex during differentiation.

**Fig. 3:**
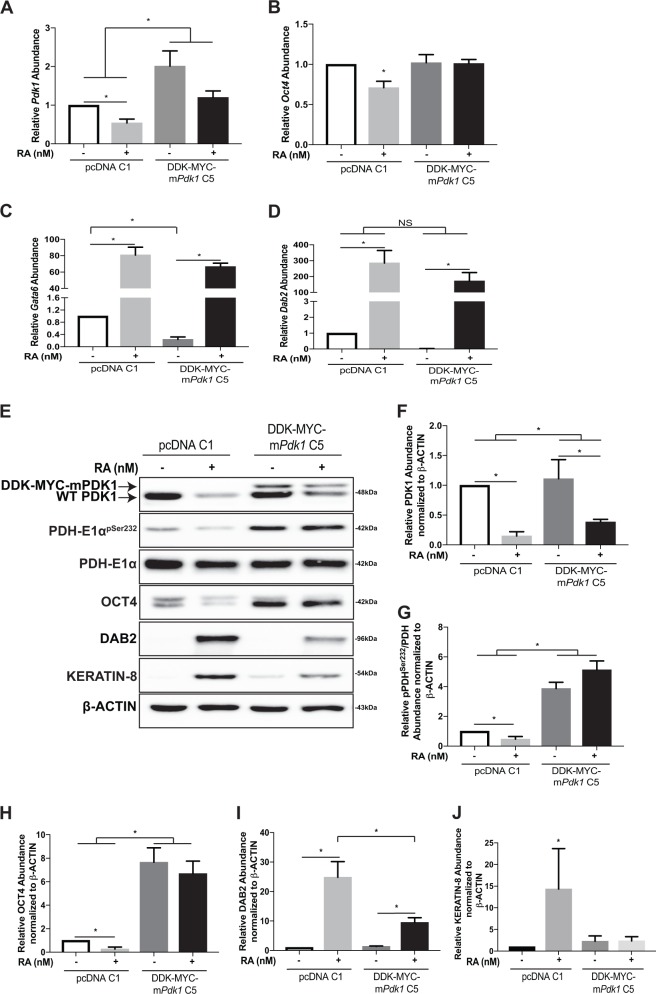
Overexpression of PDK1 attenuates RA-induced differentiation of late-passage F9 cells. qRT-PCR of relative abundance of **A** *Pdk1*, **B** *Oct4*, **C** *Gata6*, and **D** *Dab2* in pcDNA 3.1 clone 1 (pcDNA C1) or DDK-MYC-mPDK1 clone 5 (DDK-MYC-mPDK1 C5)-overexpressing F9 cells treated with DMSO or RA for 96 h. *L14* was used a constitutive gene for qRT-PCR. Representative immunoblot (**E**) and densitometric analysis of **F** PDK1, **G** PDH-E1α^pSer232^, **H** OCT4, **I** DAB2, and **J** KERATIN-8 in pcDNA C1 or DDK-MYC-mPDK1 C5-overexpressing F9 cells treated with DMSO or RA for 96 h. β-Actin served as a loading control. Values are presented as mean ± SEM of at least three biological replicates. Significance was tested using a one-way ANOVA followed by a Tukey’s test. ^*^P < 0.05

To further examine the role of PDK1 in PrE differentiation, *Pdk1*-expressing stable lines were generated (Fig. 3). As expected, clones overexpressing PDK1 showed significantly higher levels of *Pdk1* expression (Fig. 3A) and protein levels (Fig. 3E, F) over controls. To test the functionality of the exogenous PDK1, PDH-E1α^pSer232^ was assayed and results show its levels were significantly higher than those in controls (Fig. 3E, G). Next, *Oct4* expression and protein levels were examined and found to be reduced significantly in control cells treated with RA (Figs. 3B, H, respectively), but neither were affected by RA in PDK1-overexpressing cells (Fig. 3B, H). Despite seeing changes in OCT4 levels between the two clones, *Gata6* expression did not differ (Fig. 3C). However, although *Dab2* expression was significantly upregulated in control PrE cells, there was no significant difference when compared to *Pdk1-*overexpressing cells (Fig. 3D). In converse, DAB2 protein levels were significantly lower when *Pdk1* was overexpressed (Fig. 3I). Lastly, overexpressing *Pdk1* attenuated RA-induced PrE differentiation as evident by the levels of KERATIN-8, which were not significantly different from controls (Fig. 3J). Together, these results indicate that the overexpression of *Pdk1*, and inactivation of the PDH complex in F9 cells, maintains pluripotency and reduces the differentiation potential.

### Passaging alters metabolism but not differentiation potential

**Fig. 4:**
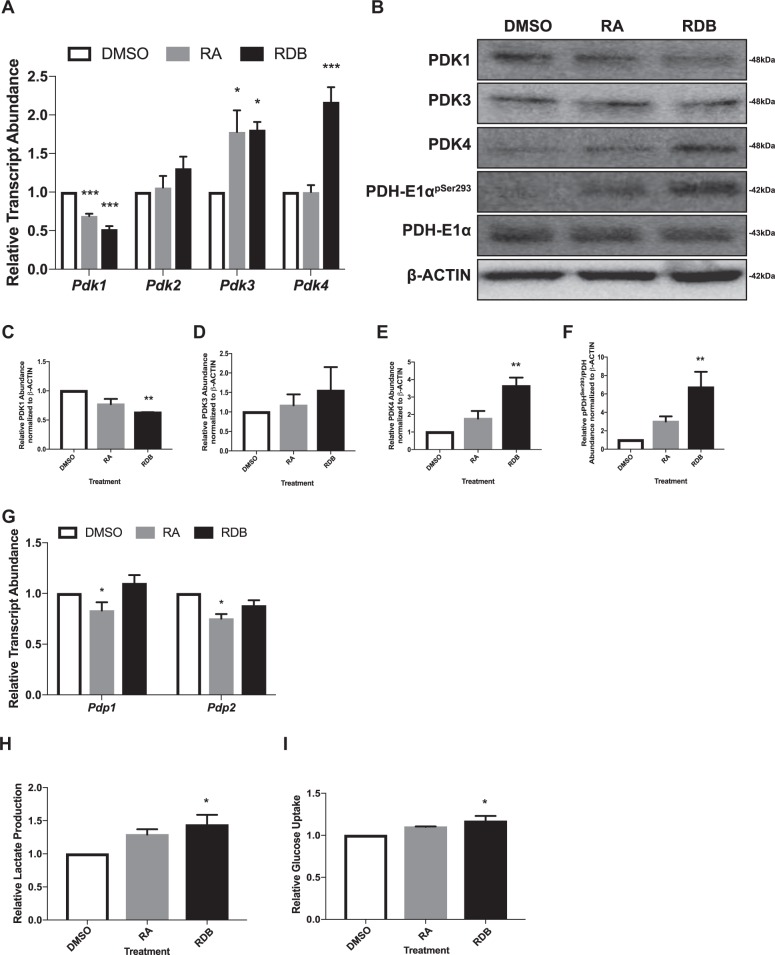
Differentiated early-passage F9 cells upregulate transcripts and enzymes involved in glycolysis. **A** qRT-PCR of *Pdk1–4* expression, representative immunoblot (**B**) of PDK1, PDK3, PDK4, PDH-E1α^pSer293^, and PDH-E1α, and densitometric analysis of **C** PDK1, **D** PDK3, **E** PDK4, and **F** PDH-E1α^pSer293^ during F9 cell differentiation. β-Actin served as a loading control. **G** qRT-PCR analysis of *Pdp1* and *Pdp2* transcripts, **H** lactate production, and **I** glucose uptake during F9 cell differentiation. *L14* was used a constitutive gene for qRT-PCR. Values are presented as mean ± SEM of at least three biological replicates. Significance was tested using a one-way ANOVA followed by a Tukey’s test. ^*^P < 0.05, ^**^P *<* 0.01, ^***^P *<* 0.001

As passage number influences stem cell quality and fate^36–40^, we asked whether F9 cells at early passages (<20) exhibited a differentiation and metabolic profile similar to that in late-passage cells. Examining markers of differentiation (*Gata6*, *Dab2*, and *Thbd*) showed similar expression profiles between the two populations (Fig. S3a-c). Similarly, OCT4 levels were higher in the undifferentiated state, and KERATIN-8 levels higher in the differentiated state (Fig. S3d-f). Thus, early- and late-passage F9 cells exhibited similar differentiation profiles, and it was expected that the metabolic profiles would be similar. To test this, the expression of *Ldh* transcripts and the levels of protein were examined (Fig. S4A and Fig. S4B, C, respectively). No significant differences in the abundance of *Ldha* and *Ldhb* were detected in early-passage cells, and at the protein level, both levels were significantly reduced by RDB (Fig. S4B, C). Differences were seen, however, in the expression of *Pdks*, and while the decreasing *Pdk1* trend with differentiation was maintained between the two populations (Fig. 4A and Fig. 2B, respectively), *Pdk3* and *Pdk4* expression was upregulated significantly only in early-passage cells (Fig. 4A). No significant difference in the abundance of *Pdk2* was evident in early-passage cells (Fig. 4A), which contrasts that seen in late-passage cells (Fig. 2B). At the protein level, PDK1 levels dropped significantly with differentiation (Fig. 4B, C), while PDK4 levels increased significantly in PE (Fig. 4B, E). Despite the increase in *Pdk3* expression (Fig. 4A), PDK3 levels remained unchanged in differentiated early-passage cells (Fig. 4B, D). These differences in PDK profiles prompted further investigation into the phosphorylation status of PDH-E1α^pSer293^, which in late-passage F9 cells decreased with differentiation (Fig. 2F, K). In early-passage cells, PDH-E1α^pSer293^ levels increased significantly, but only in PE (Fig. 4B, F). To explain the increase, PDH phosphatases (PDPs), which dephosphorylate serine residues and subsequently activate the PDH complex, were examined (Fig. 4G). Results show *Pdp1* and *Pdp2* expression was significantly downregulated in response to RA treatment, but only in early-passage cells, suggesting that the increase in PDH-E1α^pSer293^ levels is due to PDK4. To test whether the PDH complex was inactive, lactate levels in the media and glucose uptake were measured in early-passage undifferentiating and differentiated cells (Fig. 4H, I, respectively). Media from PE cells showed significantly more lactate (Fig. 4H) when compared to controls, and this was accompanied by a significant increase in glucose uptake (Fig. 4I). The relative abundance of *Glut1*–*4*, *8* and *9* in early-passage F9 cells was also examined, and while *Glut1* and *3* expression was significantly upregulated in PrE, *Glut2*, and *4* expression were only upregulated significantly in PE (Fig. S5). *Glut8* expression was downregulated significantly in PrE (Fig. S5), unlike that seen in late-passage cells (Fig. S2). Collectively, the results indicate that unlike late-passage F9 cells, those that have not been passaged extensively transition from OXPHOS metabolism toward glycolysis during differentiation.

### Mitochondrial dynamics in early- vs. late-passage cells

**Fig. 5:**
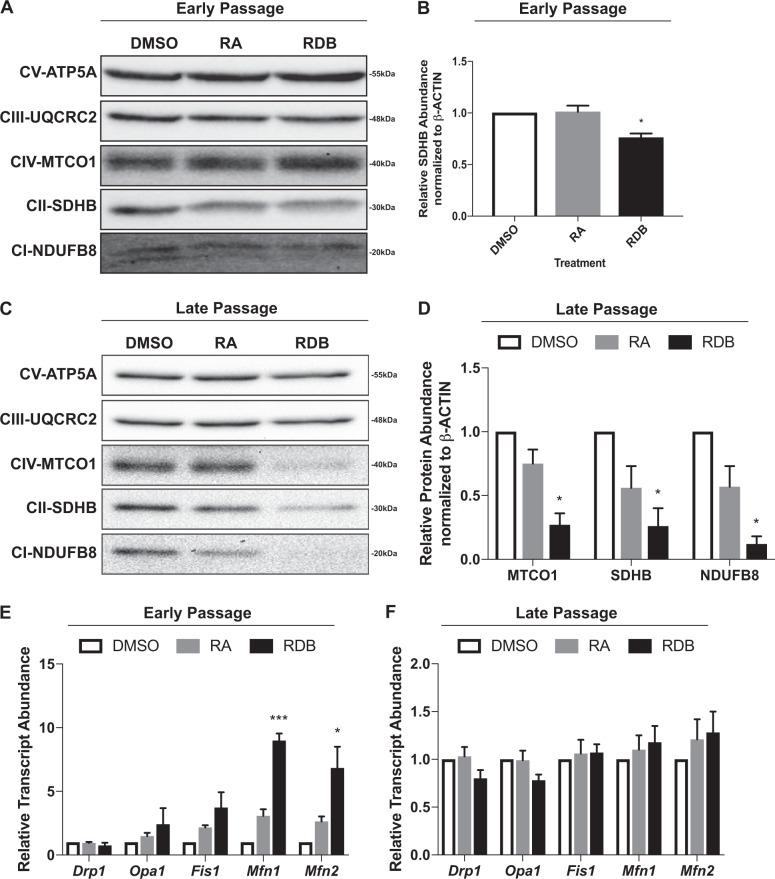
ETC components and mitochondrial dynamics are deregulated in differentiated late-passage F9 cells. Representative immunoblots and densitometric analyses of subunits in the ETC seen during the differentiation of **A**, **B** early- and **C**, **D** late-passage F9 cells. Relative transcript abundance of mitochondrial fusion proteins (*Mfn1* and *Mfn2*) and mitochondrial fission proteins (*Drp1*, *Opa1* and *Fis1*) during the differentiation of **E** early- and **F** late-passage F9 cells. *L14* was used a constitutive gene for qRT-PCR. Values are presented as mean ± SEM of at least three biological replicates. Significance was tested using a one-way ANOVA followed by a Tukey’s test. ^*^P < 0.05, ^***^P *<* 0.001

As uncoupling proteins, which have been implicated in regulating stem cell differentiation^41,42^, showed no difference between the two populations (Vorobieva and Kelly, unpublished), the focus turned to ETC proteins. Analysis revealed that undifferentiated and differentiated early-passage cells express comparable levels of all ETC subunits, with the exception of succinate dehydrogenase complex iron sulfur subunit B (SDHB) in complex II (Fig. 5A, B). Differentiation of late-passage cells to PrE or PE caused a significant decrease in the levels of MTCO1 (complex IV), SDHB (complex II), and NDUFB8 (complex I; Fig. 5C, D). In order to explain the disruption of ETC proteins, the expression profiles of genes involved in mitochondrial fission and fusion were examined. Early-passage cells showed no significant change in *Drp1*, *Opa1*, and *Fis1* expression, which encode proteins that promote mitochondrial fission; however, *Mfn1* and *2* expression was significantly upregulated in PE (Fig. 5E). No obvious changes were detected in late-passage cells (Fig. 5F), and despite seeing elevated mitochondrial ROS levels and increased activity in differentiated late-passage cells (Fig. 1A–H), the data suggests that the mitochondria in both early and late-passage cells are mature, fused and capable of OXPHOS metabolism.

### Glycolysis promotes differentiation of early-passage F9 cells

**Fig. 6:**
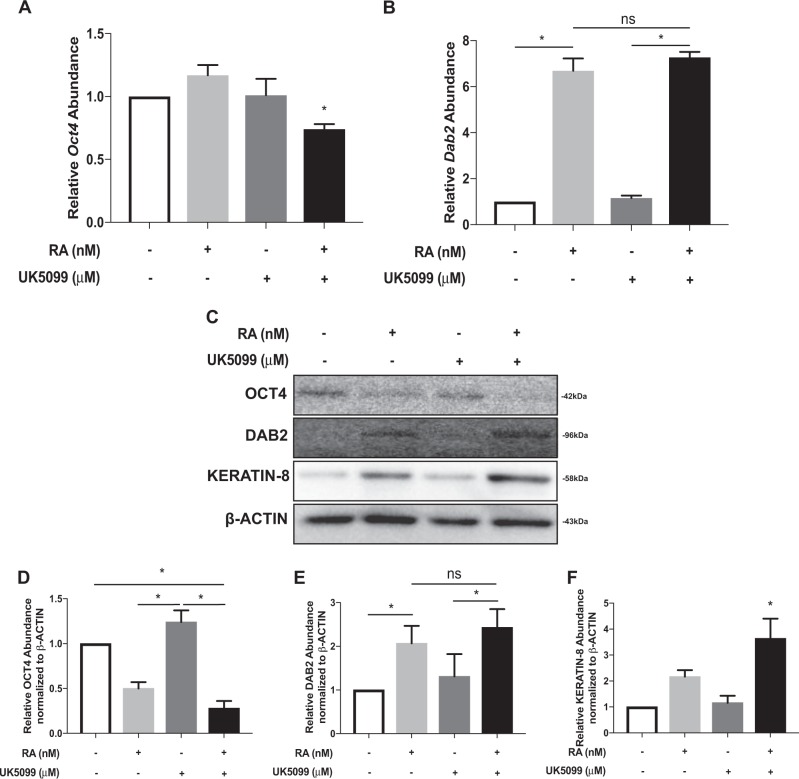
Glycolysis promotes differentiation of early-passage F9 cells. qRT-PCR analysis of **A** *Oct4* and **B** *Dab2* in F9 cells differentiated without or with UK5099 to inhibit mitochondrial pyruvate transport. *L14* was used a constitutive gene for qRT-PCR. **C** Representative immunoblot of DAB2, KERATIN-8 and OCT4, and densitometric analyses of **D** OCT4, **E** DAB2, and **F** KERATIN-8 levels in F9 cells treated with RA or RA and 50 μM UK5099. β-Actin served as a loading control. Values are presented as mean ± SEM of at least three biological replicates. Significance was tested using a one-way ANOVA followed by a Tukey’s test. ^*^P < 0.05 ns, not significant

If early-passage F9 cells transition towards glycolysis during differentiation, then promoting glycolysis in these cells should induce differentiation. To test this, early-passage cells were treated with UK5099, a non-competitive inhibitor of the mitochondrial pyruvate uptake transporter. Concentrations between 0.05 and 5 µM had no apparent effect on cell viability compared to the control (data not shown). A 5 µM concentration was selected, and results show that treatment in combination with RA caused a significant reduction in the abundance of *Oct4* relative to RA alone (Fig. 6A). Although these results suggested cells were exiting the pluripotent state, treatment alone or in combination with RA showed no additive effects on *Dab*2 expression (Fig. 6B). OCT4, DAB2, and KERATIN-8 levels were also analyzed, and as expected OCT4 levels were significantly lower in RA-treated cells, and even lower in the treatment with UK5099 (Fig. 6C, D). DAB2 levels, similar to the corresponding expression data, were not significantly different between RA or RA and UK5099 treatments (Fig. 6C, E); however, under the same conditions, KERATIN-8 levels were significantly higher (Fig. 6C, F). Thus, culturing early-passage cells under conditions to promote glycolysis enhanced the exit from pluripotency and differentiation to PrE.

### OXPHOS enhances the exit of pluripotency in late-passage cells

**Fig. 7:**
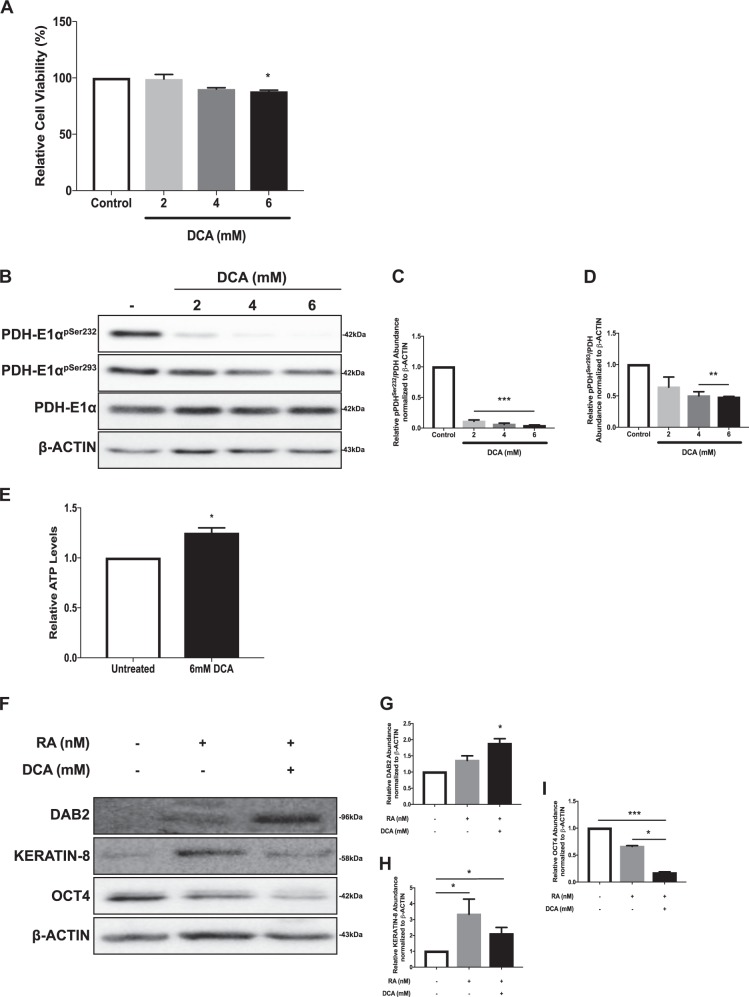
OXPHOS promotes differentiation of late-passage F9 cells. **A** MTT viability assay, representative immunoblot (**B**) of PDH-E1α^pSer232^, PDH-E1α^pSer293^, and PDH-E1α, and densitometric analyses of **C** PDH-E1α^pSer232^ and **D** PDH-E1α^pSer293^ of F9 cells treated with 0, 2, 4, and 6 mM DCA. **E** Relative ATP levels in untreated and 6 mM DCA-treated F9 cells. **F** Representative immunoblot of DAB2, KERATIN-8, and OCT4, and densitometric analyses of **G** DAB2, **H** KERATIN-8, and **I** OCT4 in F9 cells treated with RA or RA and 6 mM DCA. β-Actin served as a loading control. Values are presented as mean ± SEM of at least three biological replicates. Significance was tested using a one-way ANOVA followed by a Tukey’s test. ^*^P < 0.05, ^**^P *<* 0.01, ^***^P *<* 0.001

As promoting glycolysis in early-passage F9 cells enhanced their ability to differentiate, then promoting OXPHOS in late-passage cells should augment differentiation. To test this hypothesis, late-passage cells were cultured in increasing concentrations of DCA, a competitive PDK inhibitor, and then assayed for changes in metabolism and differentiation (Fig. 7). Cells cultured in the highest concentration of DCA (6 mM) showed a significant reduction in cell viability (88 ± 1%; Fig. 7A); however, 6 mM DCA was selected, as it caused the most significant reduction in PDH-E1α^pSer232^ and PDH-E1α^pSer293^ levels (Fig. 7B–D), resulting in significantly higher ATP levels (Fig. 7E). As for differentiation, DAB2 and KERATIN-8 levels were significantly higher than the controls, but only in cells co-treated with RA and DCA (Fig. 7F, G and Fig. 7F, H, respectively). As the results would indicate that cell differentiation was augmented by DCA, OCT4 levels in the cells should be dramatically decreased. This was tested and results show OCT4 levels were significantly reduced in RA-treated cells relative to controls (Fig. 7F, I). As expected, these levels were even more reduced in cells treated with RA and DCA (Fig. 7F, I), suggesting that promoting OXPHOS in late-passage cells not only enhanced their exit from pluripotency, but it also promoted their differentiation.

### Genomic integrity and cell cycle regulation in early- vs. late-passage cells

**Fig. 8:**
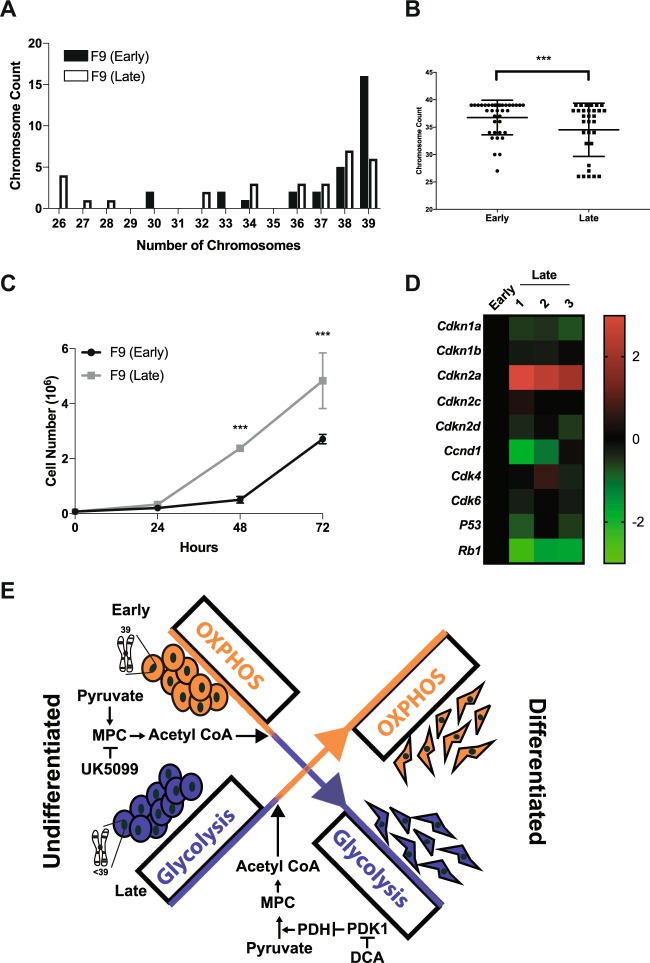
Early-passage F9 cells display a genetically stable karyotype and proliferate slower than late-passage F9 cells. **A** Number of chromosome, **B** chromosomal frequency, and **C** growth curve of early and late-passage F9 cells. **D** Differential expression of genes encoding proteins involved in cell cycle regulation and progression between early- vs. late-passage F9 cells. **E** Schematic overview of the mechanisms regulating metabolism in both populations. Values are presented as mean ± SEM of at least three biological replicates. Significance was tested using a one-way ANOVA followed by a Tukey’s test. Chromosomal distribution frequency was tested by *χ*^2^-test for the Goodness of Fit. ^***^P *<* 0.001

Although metabolic changes can influence genomic integrity^43^, reports have shown that extensive passaging of adherent cells and ESCs can induce genetic abnormalities thereby affecting cellular metabolism^44,45^. To address this, karyotyping was used to determine whether chromosomal composition could explain the variations observed between early and late-passage cells. Analysis revealed approximately 60% of the early-passage F9 cells have the proper karyotype, while less than 5% of the late-passage F9 cells had the optimal chromosome number (Fig. 8A). Furthermore, the chromosome frequency distribution of early-passage cells was significantly different from the late-passage cells (*χ*^2^ = 42.868, df = 2, *P* < 0.001, Fig. 8B). Cell proliferation was examined as this is known to be affected by chromosomal abnormalities^46^. Results showed early-passage cells proliferated at a significantly lower rate than late-passage cells (Fig. 8C). An examination of various cell cycle regulators including *Cdkn1a*, *Cdkn1b*, *Cdkn2a*, *Cdkn2c*, *Cdkn2d*, *Ccnd1*, *Cdk4*, *Cdk6*, *P53*, and *Rb1* revealed that with the exception of *Cdkn2a*, the other cell cycle markers were upregulated in late-passage cells (Fig. 8D). Together, this loss of genomic integrity could account for the differences in the proliferation rates and metabolic profiles seen between early- vs. late-passage cells.

## Discussion

Stem cells hold great potential for regenerative medicine applications; however, understanding their physiology in vitro is crucial to ensure the delivery of quality cells to patients. We used F9 cells to determine the metabolic profile during XEN-like differentiation, and how passaging may affect their metabolic profile and differentiation potential. Two cell populations were examined and although each differentiated and upregulated markers of PrE and PE (Figs. S1, 3), their metabolic profiles differed dramatically. During differentiation, late-passage F9 cells transitioned from glycolysis to OXPHOS resulting in an increase in mitochondrial ROS levels and mitochondrial activity, both correlated with increased ATP production (Fig. 1). The increase in OXPHOS during differentiation is explained by the reduction in PDK1 levels (Fig. 2F, G), resulting in reduced PDH-E1α^pSer232^ (Fig. 2F, I) and PDH-E1α^pSer293^ (Fig. 2F, K) levels. Conversely, overexpressing *Pdk1* attenuated RA-induced differentiation in these cells (Fig. 3). A similar trend occurs with iPSCs, human ESCs (hESCs), and mesenchymal stem cells (MSCs), which are glycolytic in nature due to an inactive PDH complex shuttling pyruvate to lactate^25,26^, but employ OXPHOS when differentiated^27,47,48^. The opposite scenario occurs in the mouse early embryo as naïve ESCs exhibit bivalent metabolism, while differentiated primed ESCs use glycolysis^16^, similar to differentiated early-passage cells as they increased lactate production (Fig. 4G), with elevated levels of PDK4 (Fig. 4B, E), and concomitant increased levels of PDH-E1α^pSer293^ (Fig. 4B, F). During the differentiation of adult stem cells, elevated lactate levels and subsequent increased Acetyl-CoA levels enhance histone H3K9 acetylation, a prelude required for differentiation^49^. However, this is not universal, and the fact that other stem cells transition towards OXPHOS when they differentiate^50^, highlight cell-specific differences regulated by the intricacies of the metabolome.

The metabolome is directly affected by the mitochondria^51^, whose activity is linked to substrate availability and structure^52^. ETC protein stoichiometry dictates the flow of electrons, which influences mitochondrial activity^53^. As described earlier, iPSCs upregulate glycolytic enzymes during the early stages of reprogramming^26,27^ and this coincides with the downregulation of proteins in complex I and IV, and the upregulation of proteins in complex II, III and V^54^. Although the metabolic profile of iPSCs is similar to differentiated early-passage cells, there were no detectable changes to complex I and IV in these cells (Fig. 5A, B). In contrast, the reduction in protein levels in these complexes were seen in differentiated late-passage cells, which favor OXPHOS during differentiation(Fig. 5C, D). Nevertheless, the disruption of ETC stoichiometry in late-passage cells explains the elevation in mitochondrial ROS (Fig. 1A–D), which together with cytosolic sources, activate the canonical WNT/β-catenin pathway required for F9 cell differentiation^22–24^. Surprisingly, these elevated mitochondrial ROS levels were not accompanied by changes in the levels of mitochondrial dynamic proteins (Fig. 5F) that are typically associated with mitochondria actively generating ATP^55^. Instead, an increase in the expression of *Mfn1* and *Mfn2*, which encode mitochondrial fusion-promoting proteins, was seen in differentiated early-passage cells (Fig. 5E). Similarities exist with primed ESCs and hESCs, which possess oval-shaped mitochondria with dense matrix, prominent cristae, and high mtDNA copy number, but have reduced mitochondrial respiration rates due to deficiencies in in complex I and IV^16^. These inconsistencies in the literature confounded the interpretation of our data and details to explain the differences in metabolic profile seen between early- vs. late-passaged F9 cells led us to examine other possibilities.

Genomic integrity was one possible explanation, as long-term passaging of stem cells can promote chromosomal abnormalities resulting in loss of pluripotency and low contribution to chimeras^32^. In our report, early- and late-passage F9 cells shared similar pluripotency and differentiation profiles (Fig. S1, S3); however, based on a comparison with other stem cell lines, we were unable to assign candidates that would implicate their involvement in the metabolic differences seen in the two F9 cell populations. It is known that mESC lines cultured for prolonged periods develop abnormal karyotypes, yet maintain pluripotency and differentiate when grown as embryoid bodies^33^. Our results revealed dramatic differences in the karyotypes of early- and late-passage F9 cells (Fig. 8A, B), and these would have profound effects on the physiology of each population. Differences included altered levels of ROS (Fig. 1A–D) and elevated CDKNA1 and p53 levels (Fig. 8D), the latter affecting the proliferation rate in late-passage F9 cells (Fig. 8C). Interestingly, these characteristics are seen in late-passage MSCs^47^ and hESCs^56^, and although they are attributed to abnormal karyotypes, other cell lines retain proper chromosomal composition and show no apparent change in cell proliferation rate or pluripotency potential^56^. Thus, like early- and late-passage F9 cells, stem cells have properties that may or may not change with passaging, suggesting that culturing methods and passage number are not the only factors promoting chromosomal degradation^57^.

Overall, our results clearly demonstrate that early versus late-passage F9 cells have the ability to differentiate into XEN, but they do so using different metabolic profiles (Fig. 8E). Moreover, culturing either population under conditions that favor their profile enhances their exit from pluripotency and promoted differentiation. Although several reports have documented the differentiation of F9 cells into PrE^58–63^, none have addressed the mechanisms in reference to metabolic profile or altered karyotypes that accompany extensive passaging. Thus, the desired differentiation phenotype of cells may come at a cost if genomic integrity is compromised. Furthermore, this underpins the importance of continually scrutinizing a stem cell population to ensure best practices for regenerative therapies.

## Materials and methods

### Cell culturing conditions

F9 embryonal carcinoma stem-like cells (Sigma) were cultured at 37 °C and 5% CO_2_ on 0.1% gelatin-coated plates in Dulbecco’s modified Eagle’s medium (Lonza) supplemented with 10% heat-inactivated fetal bovine serum (Thermo Fisher Scientific) and 1% penicillin–streptomycin (Thermo Fisher Scientific). All cultures were tested for mycoplasma^34^ and cells under 20 passages were classified as being early. To induce PrE differentiation, cells were treated daily for 72 h with 100 nM All-trans retinoic acid (RA; Sigma), or treated daily with 100 nM RA and 10 mM db-cAMP (RDB; Sigma) to induce PE. Cells treated with dimethyl sulfoxide (DMSO) served as a negative control.

### Generation of PDK1-stable cell line

F9 cells were plated as described above and reverse transfected using Lipfectamine 2000 (Thermo Fisher Scientific). Briefly, 200,000 cells were seeded in 35 mm gelatin-coated plates already containing 4 μg of DNA plasmid. Culture media was replaced 6 h post transfection and cells were allowed to grow for 24 h. Following incubation, media was changed and cells were selected with 2 mg/ml G418 (Gemini Bio-Products) for 2 weeks. Cells were trypsinized and seeded at low density allowing single colony formation. Single clones were selected and propagated for downstream analysis. *pcDNA 3.1* plasmid was generously provided by Dr. Robert C. Cumming and *DDK-MYC-mPDK1* overexpression construct was purchased from Origene.

### RNA isolation and quantitative reverse transcription-PCR analysis

Total RNA, isolated at 72 h from cells using a RNeasy Mini kit (Qiagen), was reverse transcribed into first strand cDNA using the High-Capacity cDNA Reverse Transcription kit (Applied Biosystems). Quantitative reverse transcription-PCR (qRT-PCR) reactions, containing 500 nM of each primer (Supplementary Table 1), SensiFAST SYBR Mix (FroggaBio), and cDNA, were carried out using a CFX Connect Real-Time PCR Detection System (Bio-Rad). Results were analyzed using the comparative cycle threshold (2^−^^ΔΔCt^) method with *L14* serving as the internal control.

### Immunoblot analysis

Protein lysates were harvested using RIPA buffer (10 mM Tris-HCl pH 8.0, 1 mM EDTA, 0.5 mM EGTA, 1% Triton X−100, 0.1% sodium deoxycholate, 0.1% SDS, and 140 mM NaCl) supplemented with HALT™ protease cocktail inhibitor (Thermo Fisher Scientific). Protein concentrations were determined using a DC™ Protein Assay (Bio-Rad) and 5–40 μg of lysates were prepared in 5X SDS loading buffer (300 mM Tris-Hcl pH 8.0, 10% SDS, 20 mM EDTA, 0.1% bromophenol blue, and 50% glycerol) and 10% β-mercaptoethanol. Proteins were separated on 5–15% polyacrylamide gels for 2 h at 100 V and then transferred onto polyvinylidene difluoride membranes (Bio-Rad) for 2 h at 250 mA and 4 °C. Membranes were placed in Tris-buffered saline with 0.1% Tween-20 (TBS-T) containing 5% w/v skim milk powder and shaken at room temperature for 30 min. Membranes were then incubated overnight at 4 °C with a primary antibody (Supplementary Table 2). After extensive washing with TBS-T, membranes were incubated with secondary antibodies (Supplementary Table 2) for 2 h at room temperature and signals were detected using a Immobilon Classico Western HRP substrate (Milllipore Sigma). Images were collected using a ChemiDoc™ Touch Imaging System (Bio-Rad); for densitometric analysis, images were analyzed using Image Lab™ (Bio-Rad).

### Karyotype analysis

Early- and late-passage F9 cells were cultured on 0.1% gelatin-coated coverslips and allowed to reach 80% confluency. Cells were treated with 0.2 μg/ml colcemid (Cayman Chemical) for 2 h at 37 °C, followed by trypsinization and suspension in pre-warmed 0.075 M KCl solution for 20 mins at 37 °C. Cells were fixed in acetic acid-methanol solution and then transferred onto pre-chilled glass slides. Chromosomes were stained with 4′,6-diamidino-2-phenylindole mounting media containing ProLong™ Gold Antifade Mountant and examined using an Axio Imager A1 microscope (Carl Zeiss). Thirty representative images were taken of each cell and chromosomes were counted manually for statistical analysis.

### Detection of mitochondrial ROS and membrane potential

Mitochondrial ROS and membrane potential were detected using MitoSOX™ Red Mitochondrial Superoxide indicator (MitoSOX; Thermo Fisher Scientific) and Tetramethylrhodamine, methyl ester perchlorate (TMRM; Thermo Fisher Scientific), respectively. Briefly, cells were seeded onto 0.1% gelatin-coated plates and allowed to differentiate as described above. At 96 h, cells were washed with phosphate-buffered saline and incubated with 100 nM MitoSOX or TMRM for 30 min at 37 °C and 5% CO_2_. Images were captured on an Axio Observer A1 Inverted microscope (Carl Zeiss) equipped with a Retiga 1300 camera (QImaging). Relative fluorescence intensity was quantified using ImageJ 1.48 V software (NIH).

### Measuring ATP levels, glucose uptake, and lactate production

ATP levels were measured using a CellTiter-Glo^®^ Luminescent Cell Viability Assay (Promega). Briefly, cells were cultured and differentiated as described above. At 72 h, cells were trypsinized, suspended in 100 µl of media, and added to a 96-well plate. After adding 100 µl of CellTiter-Glo^®^ reagent, cells were lysed for 2 min and incubated for 10 min in the dark at room temperature. Luminance was recorded using a Modulus™ II microplate multimode system (Promega) and an integration time of 1.0 s. For glucose uptake and lactate production, cells were cultured and differentiated as above, and at 72 h media was removed and centrifuged at 4 °C for 10 min at 10,000 r.p.m. Media was analyzed for glucose and lactate using a BioProfile^®^400 Chemical Analyzer (Nova Biomedical) at GCRC Metabolomics Core Facility (McGill University). Protein concentration was used to normalize values.

### Cytotoxicity assay

F9 cells were cultured in 0, 2, 4, and 6 mM dichloroacetate (DCA) and assayed for viability using a MTT (3-(4,5-dimethylthiazol-2-yl)-2,5-diphenyltetrazolium bromide) assay (Sigma). Briefly, media was removed 24 h post treatment and MTT reagent was added to cells cultured for 6 h at 37 °C and 5% CO_2_. To dissolve formazan crystals, DMSO was added to cells, which were shaken in the dark overnight at room temperature. Absorbance values at 570 nM with a reference wavelength at 650 nM were collected using the Modulus™ II microplate system described above.

### Scanning electron microscopy

F9 cells were cultured and differentiated as mentioned above. Briefly, cells were washed in 0.1 M phosphate buffer and fixed with 2% glutaraldehyde for 30 mins, followed by osmium tetroxide for 2 h at 4 °C. After fixation, cells were dehydrated in a graded series of ethanol (50, 50, 75, 85, 95, and 100%) for 5 min each. Samples were air-dried and sputter-coated with gold particles for 10 mins. Samples were viewed and images collected on a Hitachi S-3400N microscope.

### Statistical analysis

All values are presented as mean ± SEM from at least three biological replicates. Comparisons between two groups were done using a Student’s *t*-test. Comparisons between three or more groups were done using a one-way analysis of variance (ANOVA) followed by a Tukey’s honest significant difference test. Chromosomal distribution frequency was tested by *χ*^2^-test for the Goodness of Fit. All data analyses were conducted using SPSS (Version 21.0, IBM Corp.) and graphs were generated using Prism software (Version 7.0d, 2017). *P*-values were considered significant at ^*^P < 0.05, ^**^P *<* 0.01, ^***^P *<* 0.001.

## Electronic supplementary material

Supplementary Table 1

Supplementary Table 2

Supplementary Figure 1

Supplementary Figure 2

Supplementary Figure 3

Supplementary Figure 4

Supplementary Figure 5

supplementary figure legends
