# Supplementary material for: Metabolic profile and differentiation potential of extraembryonic endoderm-like cells
Source: Cell Death Discov. 2018 Sep 26;4:42. doi: 10.1038/s41420-018-0102-1 (PMC6158286; doi:10.1038/s41420-018-0102-1)
Supplement: Supplementary file 8 — supplementary figure legends [file 41420_2018_102_MOESM8_ESM.docx]

**SUPPLEMENTARY INFORMATION**

**Supplementary Fig. 1.** Late passage F9 cells differentiate into extraembryonic endoderm. **(A)** Scanning electron micrographs of undifferentiated F9 cells (DMSO), primitive endoderm (RA) and parietal endoderm (RDB). White arrows indicate differentiated cells. Expression of **(B)** *Oct4,* **(C)** *Gata6*, **(D)** *Dab2* and **(E)** *Thbd* in F9 cells treated with DMSO, RA or RDB for 72h. *L14* was used a constitutive gene for qRT-PCR. **(F)** Representative immunoblot and densitometric analyses of **(G)** OCT4, **(H)** DAB2, **(I)** KERATIN-8 and **(J)** THBD normalized to β-ACTIN during F9 cell differentiation. Values are presented as mean ± SEM of at least 3 biological replicates. Significance was tested using a One-Way ANOVA followed by a Tukey’s test. ^*^*P* < 0.05.

**Supplementary Fig. 2.** Glucose transporters expression is altered during differentiation of late passage F9 cells. Differential expression of *Glut1-4, 8 and 9* seen during the differentiation of late passage F9 cells*. L14* was used a constitutive gene for qRT-PCR. Values are presented as mean ± SEM of at least 3 biological replicates. Significance was tested using a One-Way ANOVA followed by a Tukey’s test. ^***^*P* < 0.001.

**Supplementary Fig. 3.** Early passage F9 cells differentiate to extraembryonic endoderm when treated with RA or RDB. Relative **(A)** *Gata6*, **(B)** *Dab2* and **(C)** *Thbd* expression in untreated (DMSO), primitive endoderm (RA) and parietal endoderm (RDB). *L14* was used a constitutive gene for qRT-PCR. **(D)** Representative immunoblot and densitometric analyses of **(E)** KERATIN-8 and **(F)** OCT4 abundance normalized to β-ACTIN during F9 cell differentiation. Values are presented as mean ± SEM of at least 3 biological replicates. Significance was tested using a One-Way ANOVA followed by a Tukey’s test. ^*^*P* < 0.05, ^**^*P* < 0.01, ^***^*P* < 0.001.

**Supplementary Fig. 4.** LDHA and LDHB levels are downregulated during early passage F9 cell differentiation. **(A)** Relative *Ldha* and *Ldhb* in untreated (DMSO), primitive endoderm (RA) and parietal endoderm (RDB). *L14* was used a constitutive gene for qRT-PCR. **(B)** Representative immunoblot and densitometric analyses **(C)** of LDHA and LDHB levels normalized to β-ACTIN during F9 cell differentiation. Values are presented as mean ± SEM of at least 3 biological replicates. Significance was tested using a One-Way ANOVA followed by a Tukey’s test. ^**^*P* < 0.01, ^***^*P* < 0.001.

**Supplementary Fig. 5.** Glucose transporters expression is altered during differentiation of early passage F9 cells. Differential expression of *Glut1-4, 8 and 9* seen during the differentiation of early passage F9 cells*. L14* was used a constitutive gene for qRT-PCR. Values are presented as mean ± SEM of at least 3 biological replicates. Significance was tested using a One-Way ANOVA followed by a Tukey’s test. ^***^*P* < 0.001.
